# New Poly(Ionic Liquid) Based Fiber for Determination of Oxytetracycline in Milk Samples by Application of SPME-CE Technique

**DOI:** 10.3390/molecules24030430

**Published:** 2019-01-24

**Authors:** T. Alexandra Ferreira, J. Francisco Flores-Aguilar, Eva M. Santos, Jose A. Rodriguez, Israel S. Ibarra

**Affiliations:** Área Academica de Quimica, Universidad Autonoma del Estado de Hidalgo, Carr. Pachuca-Tulancingo Km. 4.5, Mineral de la Reforma, Hidalgo 42184, Mexico; alexandrafg21@gmail.com (T.A.F.); jffa.uaeh@gmail.com (J.F.F.-A.); emsantos@uaeh.edu.mx (E.M.S.); jara.uaeh@gmail.com (J.A.R.)

**Keywords:** oxytetracycline, poly(ionic liquid), SPME, LVSS-CE

## Abstract

In this work, a procedure using solid phase microextraction in combination with capillary electrophoresis was developed for the determination of oxytetracycline in milk samples. The method involves the synthesis of poly(1-allyl-3-methyl imidazolium) chloride film on a stainless-steel bar via electropolymerization and its use as an adsorbent for oxytetracycline (OT) by an ionic exchange mechanism. The coated fiber is then immersed in milk samples for retention of oxytetracycline residues, followed by elution, drying, and reconstitution before analysis with capillary electrophoresis. The proposed method achieves a limit of detection of 70 µg L^−1^ with adequate precision and uncertainty, making this methodology appropriate for the determination of OT in milk samples. The method was applied to the pre-concentration and quantification of oxytetracycline in ten commercial milk samples. Two tested samples were positive for the presence of oxytetracycline but the concentration was below the maximum residue limit according to the international normative standard. The proposed methodology was evaluated according to the Eco-Scale approach, and the total score of 51 indicated that the methodology proposed is both green and acceptable despite the multi-stage character. SPME-CE methodology allows us to perform the sample pre-treatment and determination of OT in an effective and greener way, decreasing the number of steps during the analysis and the generation of waste.

## 1. Introduction

Oxytetracycline (OT) is a broad-spectrum antibiotic used in veterinary medicine for the prevention and treatment of different animal farm infections and it is also used in sub-therapeutic doses as an additive for growth promotion, causing weight gain in short periods of time [1,2,3]. The use of oxytetracycline may result in the presence of residual levels of it in animal-derived products, leading to great consequences in human health such as the development of antibacterial resistance, allergic affectations, gastrointestinal problems, and toxic effects. The food and drug administration (FDA) in the USA established a concentration of 300 μg L^−1^ of tetracycline (TC), oxytetracycline (OT) and chlortetracycline (CT) together as the maximum residue limit (MRL) in animal-derived products, ref. [4] while the European Union establishes an MRL of 100 μg kg^−1^ for OT [5]. Milk is a nutritious and accessible animal product, and it is thus important to determine the presence of antibiotic residues such as oxytetracycline [6].

Several analytical methods have been reported for OT analysis. Some examples include infrared spectroscopy (FT-IR) and high-performance liquid chromatography (HPLC) or capillary electrophoresis (CE) with UV-Vis, fluorescence, or mass spectrometry detection. Sample pre-treatment was identified as a critical step in the analysis of complex samples. Pre-treatment usually involves cleaning, extraction, isolation, and pre-concentration of the analyte. Pre-treatment techniques based on liquid-liquid extraction are widely used; nevertheless, there is high consumption of organic solvents. In recent years, different alternatives were developed, employing solid phase extractants. Solid phase extraction was described for the analysis of OT in milk samples employing reverse phase, ionic exchange, polymeric phases (Oasis HLB), ref. [7] and molecular imprinted polymers [8,9]. The solid phases can be also applied in solid phase microextraction (SPME), in which the extracting phase is attached on the surface of a fiber. The main advantages of SPME were the simple operation, the possibility of automation, low cost, and the possibility to analyze high sample volumes [10,11,12]. The classic sorbents used in SPME are polydimethylsiloxane, polyethyllenglycol, and cyanopropyl polysiloxane. However, there are some disadvantages, like the limited variety of commercial fibers [10]. Therefore, the development of new sorbent materials for SPME is necessary to increase the sensitivity and selectivity of the method [11].

Recently, ionic liquids (ILs) gained attention as novel sorbent materials in separation methodologies because of their nature, adsorption capacity, and selectivity towards analytes. In SPME, ILs can be immobilized by two procedures. The first is by supporting them physically onto silica or polymeric materials, so-called supported IL phases (SILPs), despite its effect on stability during the desorption step [13,14]. In order to overcome this complication, IL can be polymerized, increasing fiber lifetimes, reproducibility, and thermal-mechanical stability [11,14,15,16]. The most popular methodology to synthesize PILs is based on polymerization of imidazolium salts containing allyl or vinyl groups via free radical polymerization [17]. However, the incorporation into SMPE methods is complicated. An alternative to preparing PILs thin films is electropolymerization. This is also a radical-based method in which the polymeric film is formed onto a conductive substrate when a potential is applied to a solution containing the IL and cross-linking monomers and the radical initiator. Electropolymerization offers advantages over chemical polymerization, including the generation of uniform films and the potential for high reproducibility on a variety of electroactive surfaces [18,19].

The present work describes the design and validation of a direct immersion SPME methodology combined with capillary electrophoresis. The SPME methodology is based on the use of a stainless steel 316 bar coated with a new crosslinked copolymer of 1-allyl-3-methylimidazolium chloride and methacrylic acid as extracting phase. In order to generate a greener methodology, the polymer was obtained using electrochemical polymerization because it is considered an environmentally-friendly since the synthesis steps and the use of organic solvents are minimized [20].

## 2. Results and Discussion

### 2.1. Electropolymerization

In order to obtain the PIL film, methacrylic acid (MAA) and 1-allyl-3-methylimidazolium chloride (IL) were copolymerized in dilute aqueous sulphuric acid solutions containing potassium persulfate (K_2_S_2_O_8_) as a radical initiator. The application of a potential sweep generates the formation of free radicals, which allows the polymerization reaction to be carried out on the electrode surface according to the following reactions:

(1) $$S_{2}O_{8}^{2-}{+\;e}^{-}\;\to {\;\mathrm{SO}}_{4}^{2-}{+\;\mathrm{SO}}_{4}^{-∙}$$

(2) $${\mathrm{SO}}_{4}^{-∙}+\;\mathrm{Monomer}\;\to {\;\mathrm{Monomer}}^{∙}{+\;\mathrm{SO}}_{4}^{2-}$$

The composition of the polymer phase is particularly important to improve the extraction in SPME methodologies. In order to include a mixed mode extractant, the use of IL, MAA and EGDMA were included in the formulation. IL showed excellent selectivity by attracting anionic analytes while repelling cationic species due to their anionic-exchange properties. MAA was chosen because it was previously reported to undergo electroreductive polymerization and because of its carbonyl functionality, which is expected to form secondary bonds with metallic oxides present on the electrode surface, thereby achieving a more stable and reproducible film on the stainless-steel surface [21]. On the other hand, EGDMA was the crosslinking agent used to obtain an insoluble polymer phase and enhanced mechanical and thermal stability [11,14].

**Figure 1:**
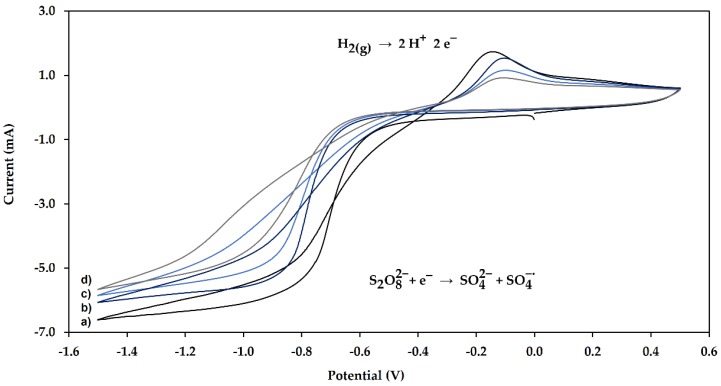
Cyclic voltammograms obtained at 100 mV s^−1^ in 7.0 mM K_2_S_2_O_8_, 5.0 mM H_2_SO_4_, 600.0 mM methacrylic acid (MAA), 100.0 mM IL, and 500 mM ethylene glycol dimethacrylate (EGDMA). (**a**) 1st scan; (**b**) 5th scan; (**c**) 10th scan; (**d**) 20th scan.

Considering the conditions described by Cram et al., the following concentrations were evaluated in this work: [K_2_S_2_O_8_] = 1.0–7.0 mM, [H_2_SO_4_] = 2.5–7.5 mM, [MAA] = 100.0–600.0 mM and [IL] = 0–100.0 mM. However, the presence of a cross-linking agent was necessary to obtain uniform films, so the use of EGDMA (500 mM) was proposed [17]. The fibers obtained were evaluated for the extraction of OT in milk samples, and the most adequate results were obtained under the following polymerization conditions: [K_2_S_2_O_8_] = 7.0 mM, [H_2_SO_4_] = 5.0 mM, [MAA] = 600.0 mM, [IL] = 100.0 mM and [EGDMA] = 500 mM [22]. Since the radical activation requires the application of a potential, cyclic voltammetry was performed. Cyclic voltammograms obtained in the experiments (Figure 1) indicate a cathodic peak at −0.8 V corresponding to the reduction of persulfate ions and an anodic peak for H_2_ oxidation. The peak positions observed are consistent with the potentials described previously for these supporting electrolytes [21]. As can be seen in Figure 1, the anodic and cathodic peak currents decrease when applying consecutive potential cycles; this indicates the formation of a passive polymer film on the working electrode surface.

**Figure 2:**
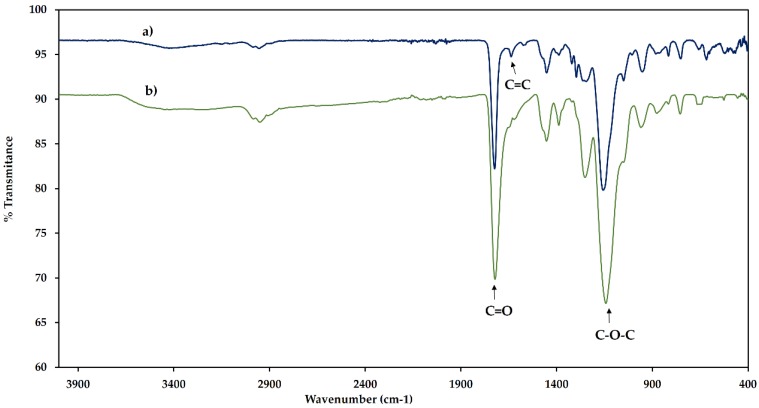
Fourier transform infrared (FT-IR) spectra of the synthesized polymers (**a**) poly(ionic liquid) (**b**) poly(methacrylic acid).

After electropolymerization, an opaque coating was obtained on the electrode surface. It was characterized using Fourier transform infrared spectroscopy (FT-IR) in order to identify the functional groups which were present in the polymeric phase. For the PIL, the spectrum in Figure 2a exhibits strong absorption bands at 1722 cm^−1^ and 1137 cm^−1^ corresponding to the presence of C=O and C-O-C groups due to the presence of EGDMA as cross-linking agent and a characteristic band at 1637 cm^−1^ due to the presence of the C=C bonds of the imidazolium ring [23]. In the case of the PMAA (Figure 2b), only the bands corresponding to the polyacrylic phase are observed. The characteristic absorption band of the C=C bonds in PIL presents low signal intensity. Therefore, in order to confirm the presence of the imidazolium ring on the polymer structure, a Raman spectroscopy was performed.

**Figure 3:**
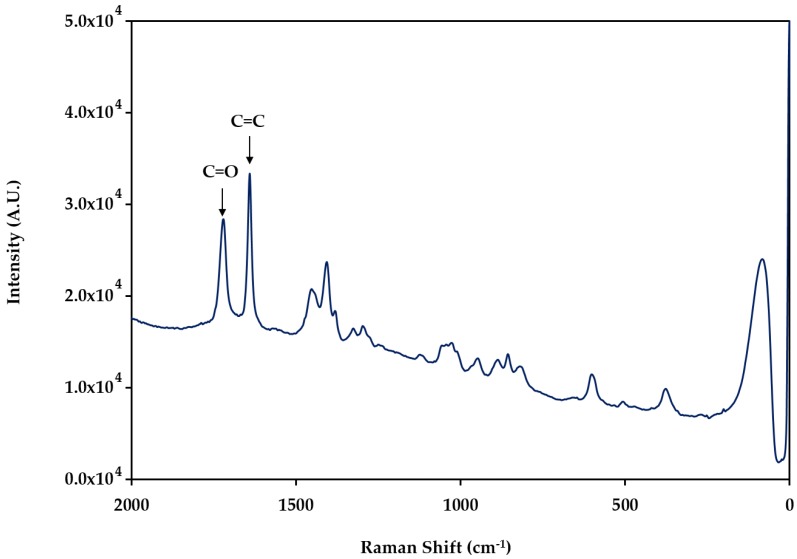
The Raman spectrum of the synthesized polymer poly(ionic liquid).

Raman spectrum of PIL (Figure 3) shows the most representative bands corresponding to C=O and C=C groups with a higher intensity, confirming the presence of the imidazolium ring of the IL and the acrylic component of the polymer.

### 2.2. Optimization of the SPME Procedure

Biological samples like milk usually contain different interferences, carbohydrates, fats, and protein components, making their characterization difficult, especially for the determination of TCs, since they are capable of forming chelate complexes with metal ions and binding with proteins [24]. Extraction and analysis of OT were tested on milk, diluted milk, and deproteinized milk samples. Milk and diluted milk samples contain ions which contribute to increase the ionic strength, affecting the conductivity of the sample and limiting the analysis with LVSS-CE. On the other hand, deproteinization employing methanol minimizes the protein interference and also decreases the solubility of the ionic components. The sample pH value affected the retention efficiency of TCs on the PIL, since is proven to be capable of interact via anion exchange. TCs present different ionic forms depending on the pH value according to the acidity constants (pKa_1_ = 3.2, pKa_2_ = 7.5, pKa_3_ = 8.9) [25]. The extraction was then evaluated on the pH interval of 2.0–10.0.

It was observed that the retention increases with the pH value, and under acidic conditions, the extraction is negligible as a consequence of the positive surface charge of the polymeric phase and the positive charge of TCs; therefore, there is electrostatic repulsion. In contrast, under alkaline conditions (pH > 7.8), TCs is negatively charged, promoting attraction with the imidazolium cation contained on the PIL. No significant differences were observed for the pH interval from 8.0–10.0, so we decided to employ pH 10.0 as the optimum extraction pH in order to ensure the ionization of the TCs [26]. Once completed, the retention fiber was immersed in 5 mL of NaOH (1.0 mM), followed by elution of TCs in acidic methanol solutions.

Since the extraction is favored in alkaline media, acetic acid 0.4 M and hydrochloric acid (0.1 and 0.2 M) were evaluated. The results showed that 0.1 M hydrochloric acid could achieve the highest recovery without increasing the ionic strength of the sample, while the methanol acidified with acetic acid (0.4 M) did not allow the effective elution of the OT and the internal standard. This confirms the existence of an anion exchange equilibrium between the Cl^−^ present on the PIL surface and the OT^−^. After the elution procedure, the extract was dried under an air stream, and 1.0 mL of deionized water was added to reconstitute the residue for LVSS-CE analysis.

### 2.3. Method Validation

Milk samples were spiked with OT concentrations of 0–750 µg L^−1^ to obtain the calibration curve. Each standard was analyzed in triplicate using the proposed methodology as described in Section 3.5. The analytical methodology was validated by determining the analytical parameters, precision, and recovery. In addition, uncertainty was measured [27,28]. The results obtained are shown in Table 1. The limit of detection (LOD) was calculated for a signal-to-noise ratio of 3.29 according to IUPAC. The LOD obtained was 70 µg L^−1^, allowing for the analysis of milk samples under MRL established by FDA and the European Union.

**Figure 4:**
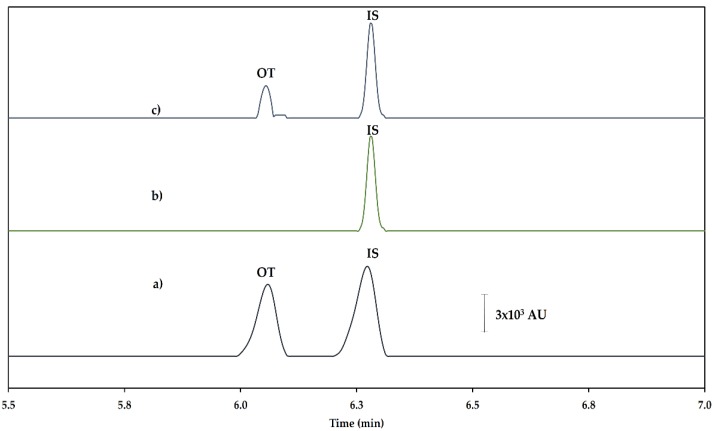
Electropherograms obtained for (**a**) the standard spiked sample in LVSS-CE (500 µg L^−1^ oxytetracycline (OT) and 500 µg L^−1^ IS), (**b**) the blank milk sample in SPE-LVSS-CE, and (**c**) the milk sample in SPE-LVSS-CE.

The electropherograms obtained are shown in Figure 4. The values obtained from the evaluation of the precision by means of inter- and intra-day repetitions are less than 10% RSD in all cases, determining that the proposed methodology presents an acceptable level of precision for the analysis of the real samples. The uncertainty value measured using a spiked sample at 250 µg L^−1^ was 0.98%, which indicates that the proposed methodology is acceptable for application to analyze OT in milk samples.

The proposed method was applied to the analysis of OT in ten different commercially available milk samples. Two samples were detected to be positive for the presence of OT, and the peaks were identified by their migration times. However, the concentrations found were below the LOQ.

Table 2 shows a comparison between different methods described for OT determination in milk. It can be seen that the described techniques present comparable LOD and precision with the proposed methodology. The high values of the relative standard deviations obtained for the methodology are associated with the concentration level employed as well as the sources of uncertainty. Despite this, the proposed methodology does not have the lowest LOD, and it is possible to satisfy the requirements for OT determination in milk samples according to MRLs established by international regulations with acceptable precision.

### 2.4. The Environmental Impact of the Methodology

In recent decades, there has been a greater awareness of the environment and the impact that chemistry has on it, making it important to implement green analytical methodologies. Green analytical chemistry considers the elimination or reduction of the use of chemical substances like organic solvents, energy consumption, waste generation, and operational risk. Since chemical analysis is a complex process consisting of several steps, it is important to evaluate the components of the methodology [33].

The Analytical Eco-Scale approach was proposed for organic synthesis and was adapted to evaluate green analytical methods [33]. In this methodology, it is assigned a score of 100 on the scale of the “greenest” analytical methodology, and for every parameter that differs from the “ideal value”, penalty points are assigned, lowering the total score of the method. The results indicate that when the total score is >75 it represents an excellent green analysis, and values >50 represent an acceptable green analysis, and values <50 indicate inadequate green analysis. Despite the multi-step character of the analytical process of the proposed methodology, it presents an analytical Eco-Scale score of 51, making our SPME-CE methodology an acceptable green analysis. The method was evaluated considering three main steps: Synthesis, SPME, and CE. The stage with the highest number of penalty points is the synthesis stage, associated with electropolishing with Cr (VI) and sulfuric acid reagents, energy consumption, and the generation of waste. However, activation of the stainless-steel surface is required to promote the formation of a homogenous polymeric phase associated with the precision of the proposed SPME-CE methodology.

## 3. Experimental Section

### 3.1. Reagents and Chemicals

All the solutions were prepared by dissolving the respective reagent in deionized water with a resistivity not less than 18.2 M Ω cm provided by a Milli-Q system (Millipore, Bedford, MA, USA). All the chemicals used were of analytical grade and were used without further purification. Oxytetracycline dehydrate (OT), doxycycline hydrate (DT), 1-allyl-3-methylimidazolium chloride (IL), sodium persulfate, ethylene glycol dimethacrylate (EGDMA), methacrylic acid (MAA), potassium dichromate, sodium hydroxide, sulfuric acid, methanol, hydrochloric acid, and acetic acid were obtained from Sigma Aldrich (St. Louis, MO, USA). EDTA sodium salt, sodium hydroxide, hydrochloric acid, sodium phosphate, and methanol were obtained from J.T. Baker (Phillipsburg, NJ USA), and 2-propanol was obtained from Fluka (St. Gallen, Switzerland). The electrolyte solution used in capillary electrophoresis consisted of 30 mM sodium phosphate, 2 mM EDTA disodium salt and 5% 2-propanol, the solution pH was adjusted to 12.0 [9].

### 3.2. Equipment

The electropolymerization experiment was performed in a potentiostat galvanostat Metrohm Autolab (model PGSTATAT302N, Amsterdam, Netherlands) with NOVA 2.0 software (Metrohm Autolab, Amsterdam, Netherlands). The working electrode was polished via electropolishing before each polymerization experiment using a DC power supply (G. W. model GPC3030D, Penang, Malaysia). Once the polymer was synthesized, it was characterized by Fourier transform infrared spectroscopy (FTIR) in a Perkin-Elmer Frontier spectrometer (Waltham, MA, USA) between 4000 and 400 cm^−11^ and Raman spectra were obtained on a BWTEK i-Raman Plus spectrometer combined with a microscope (100, 50 and 20×, a 532 nm laser excitation source (50 mW) and a HQE-CCD detector. Capillary electrophoresis was performed using a Beckman Coulter P/ACE 5500 (Fullerton, CA, USA) with a photodiode array detector (DAD). Data were acquired and analyzed using P/ACE MDQ version 2.3 software (Beckman Coulter, Fullerton, CA, USA). A pH/ion analyzer model 450 from Corning Science Products (Corning, NY, USA) was used to accurately adjust pH of solutions to 0.01 pH units.

At the beginning of each working day, the capillary was activated with 1.0 M NaOH for 15 min, followed by 0.1 M NaOH for 10 min, deionized water for 10 min, and finally the electrolyte solution for 10 min at 25 °C. The capillary was washed between analyses using 1.0 M NaOH for 4 min, 0.1 M NaOH for 2 min, deionized water for 2 min, and electrolyte solution for 4 min. The separation conditions were: A fused silica capillary (41.7 cm × 75 µm i.d.), the detector wavelength was set at 360 nm, the capillary was kept at 25 °C, and a separation voltage of 14 kV at normal polarity. Peaks were identified by migration times and co-injection of standard solutions. In order to improve the limit of detection, a large volume sample stacking capillary electrohoporesis conditions were employed using the following conditions: Injection time, 60 s; pre-concentration time, 60 s; and stacking voltage, −8 kV [31].

### 3.3. Electropolymerization

Before each electropolymerization experiment, the working electrode was electrochemical polished. A stainless steel 316 bar was immersed (exposed area 138.2 mm^2^) in an electrolyte containing a 2.5 M solution of K_2_Cr_2_O_7_ in 5 M H_2_SO_4_ and subjected to a direct electrical current of 1.5 A for 60 s at 70 °C. The stainless-steel bar is maintained anodic, with the cathodic connection being made to another stainless-steel bar.

**Figure 5:**
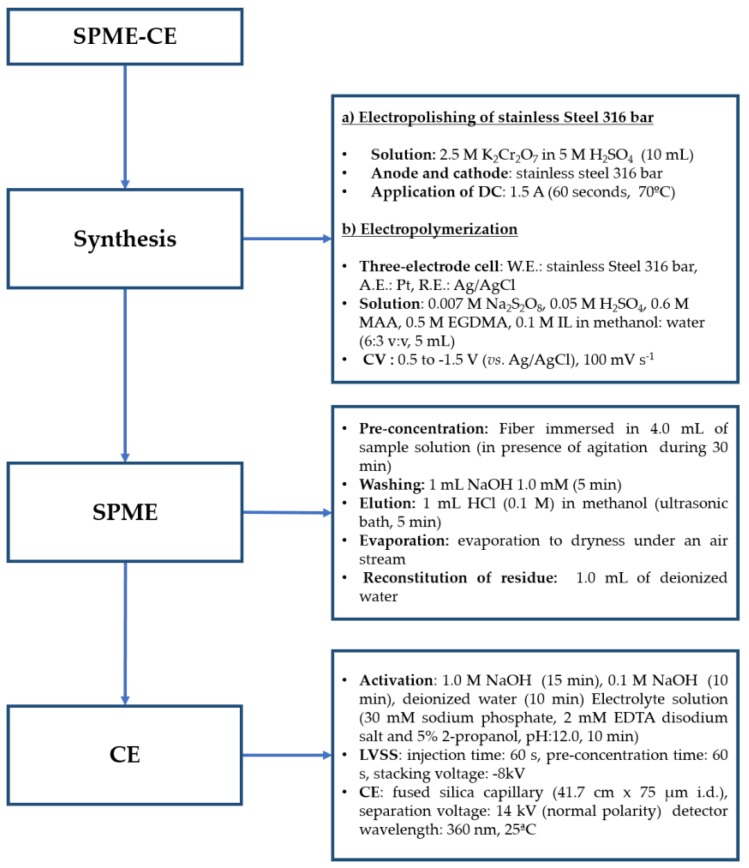
Schematic representation of the analytical procedure.

The synthesis of the adsorbent was carried out via electrochemical polymerization. A conventional electrochemical cell with a three-electrode arrangement was used, the working electrode was the stainless steel 316 bar, and platinum and Ag/AgCl were employed as the auxiliary and reference electrodes, respectively. Electropolymerization (Figure 5) was carried out using cyclic voltammetry, potentials were cycled between 0.5 and −1.5 (vs. Ag/AgCl) at a scan rate of 100 V s^−1^. The electrolyte solution consists of 0.007 M sodium persulfate, 0.05 M H_2_SO_4_, 0.6 M MAA, 0.5 M EGDMA, and 0.1 M IL in a solvent composed of methanol: Water (6:3 *v*/*v*). At the end of electrolysis, the working electrode was removed and rinsed with deionized water.

### 3.4. Sample Preparation

Ten commercially available milk samples of different brands were analyzed. Initially, doxycycline, (internal standard, 40.0 mg L^−1^) was added to 10 mL of milk. Proteins were precipitated by mixing in a vortex (1 min) 2.0 mL of milk with 6.0 mL of methanol in a polypropylene centrifuge tube (15.0 mL). The mixture was then centrifuged at 2500 rpm for 10 minutes, and 500 µL of the liquid phase was taken, the pH value was adjusted to 10.0, and the system was diluted to 10.0 mL with deionized water in a volumetric flask.

4.0 mL of the resultant solution was placed in a beaker, and the fiber was immersed in the solution. After 30 min of magnetic agitation, the fiber was then washed by submerging it in 1.0 mL of NaOH 1.0 mM (5 min). The analyte and the internal standard were eluted by immersion of the fiber in 1.0 mL of methanol acidified with HCl (0.1 M) in an ultrasonic bath for 5 min. The solution obtained was evaporated to dryness under an air stream, and the residue was reconstituted in 1.0 mL of deionized water to be analyzed in LVSS-CE.

### 3.5. Method Validation

Under optimum experimental conditions, the analytical parameters for OT applying the SPME-LVSS-CE method were obtained using a calibration curve. The calibration curve was constructed from peak area ratios (analyte: Internal standard) of spiked samples using concentrations 0–750 µg L^−1^ of OT. The precision of the method was evaluated by means of inter-day and intra-day repetitions for the peak areas obtained. The results were determined as the relative standard deviation (%RSD) obtained in the analysis of OT at two concentration levels, milk samples were spiked up to 250 and 350 µg L^−1^ OT, and the determination procedure was carried out in triplicate in three days. In order to ensure that the results obtained are a reliable source of information, the uncertainty was determined considering all the sources of uncertainty that influence the results: Sampling, calibration, recovery, precision, and limit of detection [27].

## 4. Conclusions

A sensitive, simple, and environmentally-friendly methodology for OT determination in milk using SPME-CE was developed and validated in this work. The analytical methodology consists of the use of a new IL-based adsorbent for the retention of oxytetracycline residues in commercial milk samples. The proposed methodology allows us to perform the sample pre-treatment and determination of OT in an effective way, decreasing the number of steps during the analysis and minimizing the use of organic solvents and generation of waste. The method was applied to the pre-concentration and quantification of OT in ten commercially-available milk samples with satisfactory outcomes. In order to ensure that the results obtained are a reliable source of information, uncertainty was determined considering all the sources of uncertainty that influence on the results: Sampling, calibration, recovery, precision, and limit of detection. The method was applied to the analysis of ten samples, and two were found to be positive for the presence of OT; however, the concentrations found were below the MRL according to international normative. The methodology achieved a LOD of 70 µg L^−1^ with adequate precision and uncertainty, making this methodology appropriate for the determination of OT in milk samples. On the other hand, the proposed methodology was evaluated according to the Eco-Scale approach, and the total score of 51 indicated that the methodology proposed is acceptably green despite its multi-stage character.

## Figures and Tables

**Table 1:** Validation results and uncertainty estimates employing spiked milk samples.

Parameter	Value	Parameter	Value
Concentration µg L^−1^	250	Uncertainty	
Calibration equation		Sample (u_sample_)	0.001
Analytical sensitivity ± Sb_1_	0.691 ± 0.024	Calibration (u_cal_)	0.028
Intercept ± Sb_0_	−0.028 ± 0.024	Recovery (u_true_)	0.899
R^2^	0.9962	Repeatability (u_rep_)	2.248
LOD µg L^−1^	70.36	LOD (u_LOD_)	0.280
		Combined uncertainty	0.98%
RSD (n = 3)	1.57	Expanded uncertainty	1.96%
**Result**			
Concentration ± U(*k = 2) µg L^−1^	250 ± 4.9		

*k: Coverage factor.

**Table 2:** A comparative table of analytical parameters for different determination methods described for OT analysis in milk samples.

Methodology	LOD (µg L^−1^)	%RSD (Intra day/Inter day)	Reference
HPLC-DAD	50	2.3/4.1	[6]
SPE-HPLC-DAD	113(µg kg^−1^)	4.70 (inter day)	[29]
SPE-HPLC-PDA	1.5	1.6/3.6	[30]
SPE-LVSS-CE	19	9.2/7.0	[31]
MSPE-CE	2	1.7/2.2	[32]
SPME-LVSS-CE-DAD	70 (68 µg kg^−1^)	3.5/4.5	This work
